# Combined Influence of Depression and Physical Frailty on Cognitive Impairment in Patients with Heart Failure

**DOI:** 10.3390/ijerph16010066

**Published:** 2018-12-27

**Authors:** Jong Kyung Lee, Mi Hwa Won, Youn-Jung Son

**Affiliations:** 1 College of Nursing, Dankook University, Cheonan 31116, Korea; kyunglee@dankook.ac.kr; 2 Department of Nursing, Wonkwang University, Iksan 54538, Korea; mihwon7729@gmail.com; 3 Red Cross College of Nursing, Chung-Ang University, Seoul 06974, Korea

**Keywords:** cognitive impairments, depression, frailty, heart failure, outpatients

## Abstract

Cognitive impairment is a prevalent condition and important barrier to self-care behaviors in patients with heart failure (HF). HF patients with depression or physical frailty are more likely to have reduced cognitive function. However, it remains unclear if combined depression and physical frailty increased the risk of cognitive impairments among HF populations. This study aimed to identify the influence of combined depression and physical frailty on cognitive impairments in HF. This cross-sectional study was included 289 patients with HF in outpatient cardiology clinics at a tertiary care university hospital in Cheonan, South Korea. We obtained patients’ characteristics including depression, physical frailty, and cognitive function with Korean validated tools using a face-to-face interview. The prevalence rate of cognitive impairment was approximately 27.3% in HF outpatients. We found that the combined influence of depression and physical frailty increased the risk of cognitive impairments in both unadjusted (odds ratio (OR) 4.360; 95% confidence interval (CI) (2.113, 8.994)) and adjusted models (OR 3.545; 95% CI (1.448, 8.681)). Our findings highlight that healthcare professionals need to be more aware of the vulnerable population who suffer from both depression and physical frailty at the same time. Future prospective studies should examine the causal relationships among depression, physical frailty and cognitive impairment during the HF illness trajectories.

## 1. Introduction

Heart failure (HF) as a progressive and debilitating disease is associated with adverse health outcomes and is the leading cause of hospital readmission and mortality worldwide [1,2]. Because of population aging and improved survival from coronary artery disease (CAD), the prevalence of HF is expected to double within the next 40 years, with expanding health care costs [3]. One of the important health issues for patients with HF is cognitive impairment, which is estimated to occur in between 25% and 80% of patients [1,4,5]. Several studies have shown that HF patients had lower performance in cognitive tests than individuals without HF [1,6]. People with cognitive impairment frequently exhibit reductions in psychomotor speed, working memory, control of attention and executive functions such as making plans and engaging in self-care behaviors [1,7]. For this reason, cognitive impairment in HF can negatively affect symptom relief, adherence to therapeutic regimens, quality of life, and clinical outcomes [1,8]. Despite its high prevalence and negative consequences, cognitive impairment in HF patients is usually underestimated by patients and healthcare professionals [9,10].

A recent meta-analysis of 27 studies reported that the prevalence rate of depression in HF patients ranges from 9% to 60% [11]. Furthermore, in several studies on HF and cognitive impairment, depressive symptoms were significantly associated with cognitive impairment, providing an impetus for further study into the role of depression in cognitive impairment in HF [12,13]. Frailty is also a highly prevalent condition in older HF patients; this is an important biologic syndrome characterized by low level of physical activity, slowness, weight loss, weakness, and exhaustion [14,15]. A number of studies have demonstrated high prevalence rates of physical frailty in HF and associated worse clinical outcomes such as falls, reduced mobility, hospitalization, and death [16,17]. Until now, physical frailty is well recognized as a risk factor for dementia in community dwelling older adults [18,19]. Only one cohort study recently reported that frailty is associated with cognitive impairment in HF [6].

The magnitude of risk associated with combined depression and physical frailty may be greater than the magnitude associated with individual depression and physical frailty in HF patients. While the associations of cognitive impairments with depression or physical frailty in HF patients are recognized [12,17], few studies have examined the associations of these three factors together among HF patients. Moreover, there is sparse observational research for HF population examining the impact of combined depression and physical frailty on cognitive impairment. Therefore, the purpose of this study was to determine combined influence of depression and physical frailty on cognitive impairment in patients with HF.

## 2. Materials and Methods

### 2.1. Study Design and Sample

This study adopted a cross-sectional research design. Patients with HF were recruited from the outpatient clinic of a tertiary care teaching hospital in South Korea between December 2016 and April 2017. HF was diagnosed by a cardiologist, an expert in HF, based on clinical history including HF symptoms and somatic signs such as the level of B-type natriuretic peptide, pulmonary rales, and increased pressure in the jugular veins. Moreover, the left ventricular ejection fraction (LVEF) was assessed using transthoracic echocardiography findings. This study included patients with HF with preserved (normal EF) or reduced EF. Participants were all outpatients 40 years and older who received care for a verified diagnosis of HF. Exclusion criteria were any kind of end-stage disease resulting in estimation of life expectancy of less than six months, and a history of paralysis or physical disabilities. Also patients were excluded if they had suffered from a psychiatric illness including major depressive disorders before they were diagnosed with HF.

Of the 300 outpatients who were asked to participate, 11 patients refused to participate because of lack of time and understanding of the objectives and purpose of this study. The other 289 outpatients participated and completed the survey. G-Power 3.1 was used to estimate the power of this study by selecting a logistic regression as the statistical analytical method [20]. A total of 243 patients were needed for a two-tail logistic regression analysis at an α level of 0.05 and a β level of 0.10 (90% power) and for a 1.7 odds ratio (OR), based on previous results [4,6]. Consequently, a sample size of 289 patients was considered an adequate sample size.

### 2.2. Measurements

#### 2.2.1. Socio-Demographic and Clinical Related Characteristics

Socio-demographic characteristics including age, gender, education, marital status, occupation, and monthly income were assessed using a self-made questionnaire with face-to-face interviews. In Korea, those with a monthly income of less than 1,000,000 Korean won (KRW) are basic livelihood security recipients according to the national basic livelihood security program.

Clinical information was obtained from electronic medical records and included the New York Heart Association (NYHA) functional classification, duration of HF diagnosis, physician-diagnosed comorbidities (hypertension, diabetes, CAD, atrial fibrillation, etc.), LVEF (%), body mass index (BMI, kg/m^2^), systolic and diastolic blood pressure (SBP, DBP, mmHg), hemoglobin (Hb, g/dL), and prescribed medication (aspirin, angiotensin converting enzyme inhibitor, angiotensin receptor blocker, and diuretics).

#### 2.2.2. Depression

We adopted the Korean version of the Patient Health Questionnaire-9 (PHQ-9) after obtaining permission from the authors of the instrument [21]. The PHQ-9 has been proposed as a valuable tool for evaluating depressive symptoms among HF patients [22]. Park et al. [21] formally translated the PHQ-9 into Korean and performed standardization of this Korean version, which is a self-administered questionnaire assessing nine depressive symptoms [23]. The scale has nine items, which are based on the Diagnostic and Statistical Manual of Mental Disorders, 4th Edition (DSM-IV) criteria. There are four answer options: not at all (0), several days (1), more than half of the days (2), and nearly every day (3). The sum score ranges from 0 to 27 scores. Higher scores indicate a higher level of depressive symptoms. PHQ-9 scores ≥ 10 had a sensitivity of 88% and a specificity of 88% for detecting major depression [24,25,26]. Thus, patients with the PHQ-9 scores ≥10 were attributed to depression group and the PHQ scores 0–9 were attributed to non-depression group in this study. The Cronbach’s alpha for our sample was 0.84.

#### 2.2.3. Physical Frailty

Physical frailty was measured using the Korean version of the FRAIL (Fatigue, Resistance, Ambulation, Illnesses, and Loss of weight) scale after obtaining permission from the authors of the instrument [27] based on the original FRAIL scale [14]. The FRAIL scale is well validated and considered reliable for cardiovascular disease [28]. Regarding five items, a score of 1 indicates its presence, and a score of 0 indicates its absence. This scale focuses on physical performance and classifies patients according to whether or not they are able to perform a task. K-FRAIL scores range from 0 to 5 (i.e., 1 point for each component; 0 = best to 5 = worst) and represent frail (3–5), pre-frail (1–2), and robust (0) health status. The researchers considered participants to be frail if they scored a total of 3 or more in these domains (i.e., FRAIL scale ≥3) based on previous studies [28,29].

#### 2.2.4. Cognitive Function

Cognitive function was assessed with a validated Korean version of the Mini-Mental Status Examination (MMSE-K) by Park and Kwon [30] through interviews with a trained interviewer. We adopted this instrument after *obtaining* permission from the authors of the instrument. The MMSE, developed by Folstein et al. [31], is widely used as a brief screening test for assessing cognitive function in patients with HF [13]. The MMSE has a good reliability, internal consistency and test-retest reliability for moderate to severe cognitive impairment. The MMSE consists of 30 items; it includes tests of orientation (10 points), short-term memory registration and recall (6 points), attention (5 points), naming (2 points), following verbal commands (4 points), judgment (2 points), and copying a double pentagon (1 point). MMSE scores range from 0 to 30, and lower scores indicate poorer cognition. The traditional MMSE cut-off score for cognitive impairment is 23/24 [31]. In this study, a cut-off score of 27 for cognitive impairment was used in the main analyses for the following reasons. First, according to some studies using an HF population, there is evidence that a score ≥28 indicates normal cognitive function, 25–27 indicates mild cognitive dysfunction and ≤24 indicates cognitive dysfunction with a high risk of dementia [1,8,32]. Second, the participants in this study were relatively more educated based on the mean age of 62.84 years; 69.6% of the participants graduated from middle school, as compared with the Korean HF sample [2]. The standard cut-off score of 23/24 would not provide adequate classification for highly educated elderly persons [33]. This evidence supported the decision to use a cut-off score of 27 for cognitive impairment in this study.

### 2.3. Ethical Consideration and Data Collection

This study was approved by Dankook university hospital institutional review board in Cheonan (IRB No 2016-10-013). All eligible patients were first approached by the research nurse. Written informed consent was obtained from patients who agreed to participate in the study at the outpatient clinic visit. This information included details about the study aim, confidentiality, and anonymity of the information, and voluntary participation. This study complies ethically with the Declaration of Helsinki.

### 2.4. Data Analysis

Collected data were analyzed using the Statistical Package for the Social Sciences (SPSS) version 23 (SPSS Inc., Chicago, IL, USA). Descriptive statistics were used to summarize data for presenting patient characteristics. Kolmogorov–Smirnov was used to examine the normal distribution of variables. Continuous data were presented as the mean ± standard deviation and categorical data were presented as numbers with percentages. Differences in continuous variables were tested using an independent *t*-test, and differences in categorical variables were assessed using the Pearson *χ*^2^ test. Multiple logistic regression was used to identify the influence of individual and combined depression and physical frailty on cognitive impairment after adjusting for patients’ baseline characteristics, which were significantly related to cognitive impairment in univariate analysis. We assessed multicollinearity for all independent variables using variance inflation factor (VIF) and tolerance values. Logistic regression models were used to compute odds ratios (ORs) with 95% confidence intervals (CIs). A *p*-value <0.05 was used for all tests to indicate statistical significance.

## 3. Results

### 3.1. Patient Characteristics

The patients’ characteristics are shown in Table 1. The mean age in this study was 62.84 (±11.81) years. 62 patients (21.5%) were female, and 88 patients (30.4%) had elementary school education or below. In terms of clinical characteristics, 33 patients (11.4%) were NYHA classification III–IV and the mean duration of HF diagnosis was 4.54 (±48.61) years. Two hundred and seven patients (71.6%) had comorbid CAD and mean LVEF % for all patients was 42.79 (±9.05). Seventy-nine patients (27.3%) with HF had cognitive impairment with a mean score of 24.64 (±2.69) using the MMSE-K.

### 3.2. Depression and Physical Frailty according to Cognitive Function Status in HF

Table 1 shows that patients with cognitive impairment were significantly older, predominantly female, less educated, single, and unemployed with monthly income of less than 1,000,000 KRW than patients without cognitive impairment. In addition, patients with cognitive impairment had significantly higher proportions of NYHA III–IV and diuretic use, less comorbid CAD, and lower BMI and Hb than patients without cognitive impairment. With regard to the level of depression and physical frailty, patients with cognitive impairment were relatively more depressed (4.94 ± 4.00) and frail (2.29 ± 1.22) than patients without cognitive impairment.

### 3.3. Prevalence of Cognitive Impairment According to Depression and Physical Frailty

Table 2 presents the prevalence of cognitive impairment according to depression and physical frailty status and their relationship. Patients with depression (*n* = 82, 28.4%) had significantly higher proportions of being frail than patients who were not depressed (*p* < 0.001). Also, patients with depression had a significantly higher prevalence of cognitive impairment than those who were not depressed (*p* = 0.009). With regard to frailty, patients who were physically frail (*n* = 67, 23.2%) had significantly higher prevalence of cognitive impairment than those who were robust or pre-frail (*p* < 0.001) (Table 2).

### 3.4. Association of Depression and Physical Frailty with Cognitive Impairments in HF

We performed multiple logistic regression analysis to assess the association of individual or combined depression and physical frailty with the prevalence of cognitive impairment after adjustment for confounding factors (Table 3). Patients who were depressed and frail had higher likelihoods of the presence of cognitive impairment in both an unadjusted model (OR 4.360; 95% CI (2.113, 8.994)) and a model adjusted for the significant factors identified in the univariate analyses (OR 3.545; 95% CI (1.448, 8.681)).

## 4. Discussion

In this study, the prevalence rate of cognitive impairment in HF patients was 27.3%, estimated using the Korean version of MMSE, which was consistent with previous studies that used this measure in patients with HF [3,6,8]. On the other hand, our result was relatively low compared to Cameron et al.’s [32] finding of 73% for hospitalized HF patients. Some studies with other screening method such as the Montreal Cognitive Assessment (MoCA) for cognitive impairment have shown different results, ranging from 15.4% to 41% [1,9]. In a comparative study of the MoCA and MMSE in a small sample of patients, Hawkins et al. [10] reported that the MMSE was slightly better in terms of sensitivity and specificity than the MoCA. As a consequence, we employed a validated Korean version of the MMSE. The MMSE is widely used as a frontline screening for mild cognitive impairment or dementia [5,7]. In addition, we adopted a cut-off score of 27 for cognitive impairment in HF based on previous studies [8,32]. Unlike our study, several studies using MMSE in patients with HF adopted a score of 24 and below for cognitive impairment [15,34]. Future studies are needed to demonstrate the optimal screening method and recommended cut-off score for cognitive impairment in HF populations based on various settings and cultural differences.

Our study showed that individual prevalence rates of depression (≥10) and physical frailty (≥3) were approximately 28.4% and 23.2%, respectively. For prevalence of depression, this result was supported by one review paper, which reported the prevalence rate of depression as varying from 19.3% to 33.6% based on diagnostic interviews and questionnaires with different measures [11]. Moreover, the prevalence rate of depression in this study was similar to another Korean review, which found prevalence rates of 24% to 68% for prevalence of depression among Korean HF patients depending on whether the PHQ-9 cut-off scores were set at 10 [24,35]. With regard to physical frailty, our result was somewhat low compared to previous studies. This is based on a systematic literature study, which included a total of 26 studies up to 2016 [16] and reported 36.2% to 52.8% prevalence rates of frailty depending on the measure. However, the prevalence rate of physical frailty in this study was relatively high compared to the Korean elderly population without HF, which was determined to be 17.5% by the K-FRAIL scale [27]. This implies that HF patients may have higher prevalence of physical frailty than those without HF. Interestingly, our study showed that the prevalence rate of co-existing depression and physical frailty was around 13.5%. However, the co-existing prevalence rate is not considerably higher than individual prevalence rate of either depression or physical frailty. This combined prevalence can avoid overestimating the value from the individual prevalence of depression and physical frailty due to overlapping symptoms.

Most importantly, the strength of our study was that this is the first study to describe the influence of depression and frailty on cognitive impairment in HF population. We observed that patients with both depression and physical frailty showed the highest risk of cognitive impairment after adjusting for confounders in comparison to those who were only depressed or frail. This result is in line with findings from prior studies that reported a significant relationship between depression, physical frailty, and cognitive impairment in an older people without HF [18,19] and depression and physical frailty in HF each as a significant predictor of cognitive impairment in prospective cohort studies [4,36]. Predictably, the two conditions are highly comorbid, but the reasons for their co-occurrence are unclear [19]. In univariate analysis, we observed that the patients who had depression (≥10) had significantly higher prevalence of being frail (≥3) than those who were not depressed. A prospective study of the relationship between depression and frailty suggested that depression might increase the risk of frailty [35]. Specifically, depression may predict indicators of frailty due to the decrease in social ties, gait speed, and physical activity, or due to the increase in sedentary lifestyle [17]. On the other hand, frailty is a geriatric syndrome resulting from age-related cumulative declines, which could be influenced by pain, mobility and balance problems, and weakness. These risk factors may also cause disability or functional decline and thus lead to depression [19]. To date, there are still many controversies regarding the overlapping symptoms and the bidirectional relationship between depression and frailty [19,37]. Therefore, there is a need to critically examine all aspects of depression and physical frailty in HF, including standardizing the measurement of depression and physical frailty in HF, understanding the underlying pathological mechanisms, and mitigating the individual and combined impacts of depression and physical frailty on cognitive impairment in HF.

The severity and breadth of cognitive impairments in HF are likely because of intermittent cerebral hypoperfusion and overstimulation of the sympathetic nervous system [38]. Thus, further studies are also needed to investigate the relationships among neurohormonal and autonomic nervous system dysfunction of HF, depression, frailty and cognitive impairment. 

The high morbidity and mortality associated with depression, frailty, and cognitive impairment indicate that it should be a priority for future research to determine strategies to improve patient outcomes, enhance health related quality of life, and lower health care costs in this growing population. Moreover, this study highlights a more comprehensive and multidimensional approach to the care of older HF patients at high risk for cognitive impairment.

## 5. Limitation

There are several limitations to the present study. Firstly, our study was not designed to evaluate longitudinal associations between depression, physical frailty, and cognitive function changes in HF patients. The severity of depression, physical frailty, and cognitive impairments in the HF population may change over time, especially in elderly patients. Therefore, future studies are needed to determine the optimal screening intervals for these three factors to prevent adverse outcomes, including cognitive decline during the HF disease trajectory. Secondly, because all participants were from a single medical center, the results are limited in terms of generalizability. Lastly, the present study included a small portion of the NYHA functional classes III and IV. Higher NYHA *classes* are positively associated with cognitive dysfunction in patients with HF. Therefore, future studies should include all four categories of NYHA with in sufficient and similar proportion.

## 6. Implications for Future Research and Practice

Despite the important knowledge gained from cross-sectional studies on these topics, using a prospective research design can aid in focusing on individual or combined long-term impacts of depression and physical frailty on cognitive impairments and their association with changes in mental well-being. Our findings suggest that a careful assessment for depression and physical frailty in HF is necessary for preventing cognitive impairments in healthcare settings. Moreover, a multidisciplinary intervention programs for reducing depression and physical frailty, for example including regular walking or peer group support, should be designed and provided for preventing cognitive impairment in patients with HF. Also, collaboration among healthcare professionals can lead to innovative depression, physical frailty and cognitive function screening and treatment initiatives for improvement of self-care among HF patients with older adults.

## 7. Conclusions

Our data showed that approximately one-fourth of HF patients had cognitive impairments, depression, and physical frailty. Most importantly, this study highlights that the presence of co-existing depression and frailty is associated with cognitive impairment in HF patients. Therefore, early detection of the coexistence of depression and physical frailty as well as depression or physical frailty alone may help to prevent cognitive decline and improve clinical outcomes in HF patients. Prospective studies are needed to clarify possible mechanisms for the association between depression, physical frailty, and cognitive function in HF populations.

## Figures and Tables

**Table 1:** Comparison of patients characteristics, depression, frailty, and cognitive function among heart failure patients with and without cognitive impairment (*n* = 289).

Characteristics	All Patients (*n* = 289)	Without Cognitive Impairment (*n* = 210)	With Cognitive Impairment (*n* = 79)	*p*
	*n* (%) or Mean ± SD	*n* (%) or Mean ± SD	*n* (%) or Mean ± SD	
Age (years)	62.84 ± 11.81	59.42 ± 10.72	71.95 ± 9.57	<0.001
Gender, women	62 (21.5)	30 (14.3)	32 (40.5)	<0.001
Education, elementary or below	88 (30.4)	36 (17.1)	52 (65.8)	<0.001
Marital status, married	227 (78.5)	175 (83.3)	52 (65.8)	0.002
Occupation, yes	140 (48.4)	129 (61.4)	11 (13.9)	<0.001
Monthly income, <1,000,000 KRW	131 (45.3)	71 (33.8)	60 (75.9)	<0.001
NYHA, III–IV	33 (11.4)	20 (9.5)	13 (16.5)	0.109
Duration of HF diagnosis (years)	4.54 ± 48.61	4.52 ± 44.17	4.74 ± 59.65	0.728
Hypertension, yes	133 (46.0)	93 (44.3)	40 (50.6)	0.356
DM, yes	80 (27.7)	57 (27.1)	23 (29.1)	0.769
CAD, yes	207 (71.6)	158 (75.2)	49 (62.0)	0.029
AF, Yes	10 (3.5)	8 (3.8)	2 (2.5)	0.733
LVEF (%)	42.79 ± 9.05	43.32 ± 9.06	41.39 ± 8.96	0.107
BMI (kg/m^2^)	24.37 ± 3.12	24.66 ± 2.98	23.61 ± 3.36	0.016
SBP (mmHg)	128.86 ± 18.16	128.66 ± 17.00	129.41 ± 21.10	0.756
DBP (mmHg)	79.58 ± 12.63	80.45 ± 12.21	77.28 ± 13.50	0.070
Hb (g/dL)	13.57 ± 1.71	13.92 ± 1.62	12.64 ± 1.57	<0.001
Aspirin, yes	240 (83.0)	174 (82.9)	66 (83.5)	0.999
ACEI/ARB, yes	123 (42.6)	87 (41.4)	36 (45.6)	0.882
Diuretics, yes	100 (34.6)	65 (31.0)	35 (44.3)	0.038
Depression (PHQ-9 score)	3.69 ± 3.61	3.22 ± 3.35	4.94 ± 4.00	<0.001
Physical frailty (the Frail score)	1.86 ± 1.02	1.71 ± 1.01	2.29 ± 1.22	0.001
Cognitive function (MMSE-K)	27.95 ± 2.56	29.20 ± 0.77	24.64 ± 2.69	<0.001

KRW = Korean won; NYHA = New York Heart Association; HF = heart failure; DM = diabetes mellitus; CAD = coronary artery disease; AF = atrial fibrillation; LVEF = left ventricular ejection fraction; BMI = body mass index; SBP = systolic blood pressure; DBP = diastolic blood pressure; Hb = hemoglobin; ACEI = angiotensin converting enzymes inhibitors; ARB = angiotensin receptor blocker.

**Table 2:** Prevalence and associations of depression and frailty with cognitive impairment (*n* = 289).

Variables	Categories (*n*)	Not Depressed (*n* = 207)	Depressed (*n* = 82)	*p*	Robust or Pre-frail (*n* = 222)	Frail (*n* = 67)	*p*
		*n* (%)	*n* (%)		*n* (%)	*n* (%)	
Frail status	Robust or pre-frail (*n* = 222)	179 (86.5)	43 (52.4)	<0.001			
	Frail (*n* = 67)	28 (13.5)	39 (47.6)				
Cognitive function	Without cognitive impairment (*n* = 210)	160 (77.3)	50 (61.0)	0.009	173 (77.9)	37 (55.2)	<0.001
	With cognitive impairment (*n* = 79)	47 (22.7)	32 (39.0)		49 (22.1)	30 (44.8)	

**Table 3:** Association of depression and frailty with cognitive impairment (*n* = 289)**.**

Predictors (*n*)	Unadjusted			Adjusted *		
	OR	95% CI	*p*	OR	95% CI	*p*
Non-depression and non-frailty (179)	1			1		
Depression alone (43)	1.326	0.611–2.879	0.476	1.635	0.659–4.054	0.289
Frailty alone (28)	1.770	0.741–4.226	0.198	0.852	0.303–2.394	0.761
Depression and frailty (39)	4.360	2.113–8.994	<0.001	3.545	1.448–8.681	0.005

* Adjusted for age, sex, education, spouse, occupation, monthly income, comorbid coronary artery disease, body mass index, hemoglobin and diuretics. OR = odds ratio; CI = confidence interval; PHQ-9 = patients health questionnaire 9.
